# Amplatzer Plug Embolization for Iatrogenic Aortic Arch Catheterization: A Rescue Technique

**DOI:** 10.1007/s00270-026-04521-4

**Published:** 2026-07-14

**Authors:** Marilena Georgiades, Felix J. Lange, Maciej Pech

**Affiliations:** Clinic for Radiology and Nuclear Medicine, University Hospital Magdeburg, Magdeburg, Germany

**Keywords:** Central venous catheterization, Arterial injury, Vascular plug

## To the Editor,

Central venous catheterization is indispensable in critical care for drug delivery, fluid resuscitation, and hemodynamic monitoring. Despite the widespread adoption of ultrasound guidance, inadvertent arterial puncture and catheter malposition still occur. Most reported cases involve supra-aortic vessels such as the carotid or subclavian arteries, whereas direct placement into the aortic arch is exceedingly rare and potentially catastrophic [1]. Conventional management strategies—including open surgical repair, stent–graft placement, or vascular closure devices—may be unsuitable in frail patients or complex anatomy. We report a rare case of inadvertent central venous catheter insertion into the aortic arch after traversing the left lung, successfully managed with endovascular plug embolization.

An 84-year-old Caucasian woman with a complex medical history, including prior Stanford type *A* aortic dissection treated with replacement of the aortic valve, aortic root, and ascending aorta, combined with reimplantation of the supra-aortic vessels without complete aortic arch replacement, presented to the emergency department with hypotension, tachycardia, dyspnea, and fever up to 38.8 °C. Her history also included persistent atrial fibrillation, hypertensive heart disease (NYHA III), multifactorial anemia, and recent INR derangement under phenprocoumon therapy complicated by spontaneous subdural and sublingual hematomas.

**Fig. 1:**
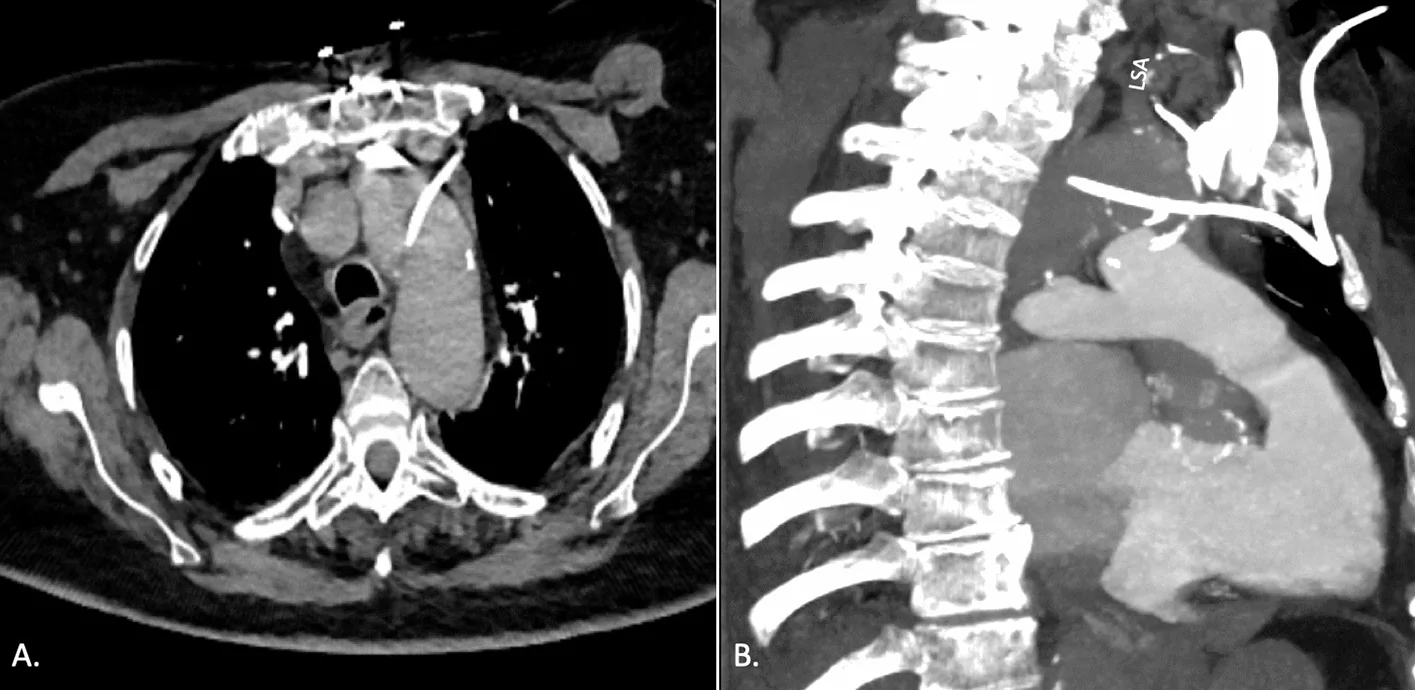
Pre-interventional CT demonstrating central venous catheter malposition in the aortic arch. Pre-interventional contrast-enhanced CT in axial (**A**) and sagittal (**B**) planes, including sagittal MIP reconstruction. The catheter tip is located within the aortic arch (**A**) after traversing the left upper lobe of the lung and entering the thoracic aorta (**B**). LSA, left subclavian artery

Due to poor peripheral venous access, a central venous catheter (CVC) was placed pre-hospital in the left subclavian region using a landmark-based technique. On admission, the patient required continuous catecholamine support. Pulsatile blood return from the catheter prompted arterial blood gas analysis, confirming arterial placement. Laboratory findings and clinical presentation were consistent with septic shock. Blood and urine cultures grew Proteus mirabilis, confirming urosepsis. CT imaging demonstrated catheter malposition within the aortic arch after traversing the left upper lobe, along with chronic postsurgical changes (Fig. 1).

The patient was stabilized in the intensive care unit (ICU) with intravenous meropenem and vasopressor therapy. Following clinical improvement and resolution of fever, management options were reassessed. Given extreme surgical risk, a minimally invasive endovascular strategy was preferred. However, standard stent–graft therapy was not feasible due to the catheter’s proximity to the reimplanted supra-aortic branches. After multidisciplinary discussion, endovascular retrieval with tract embolization was planned.

Under general anesthesia, the right common femoral artery was accessed under ultrasound guidance and a 5F sheath was placed, later upsized to a 7Fr/90 cm sheath. Through the malpositioned left pectoral 7Fr triple-lumen CVC, 0.035’’ and 0.014’’ guidewires were advanced into the descending aorta. The 0.035’’ guidewire was captured using a 20 mm Gooseneck snare via femoral access, establishing a stable through-and-through rail. This provided controlled access across the perforation and facilitated device delivery.

Over this rail, the femoral sheath was advanced across the aortic entry site while the CVC was removed, leaving the 0.014” guidewire in situ. A contralateral left femoral arterial access was obtained, and a pigtail catheter was advanced into the ascending aorta for angiographic control. Digital subtraction angiography confirmed correct device positioning and vascular anatomy, supporting the pre-procedural imaging-based decision to use a 12-mm Amplatzer Vascular Plug (Abbott Medical, USA), which was intentionally oversized to ensure secure sealing.

**Fig. 2:**
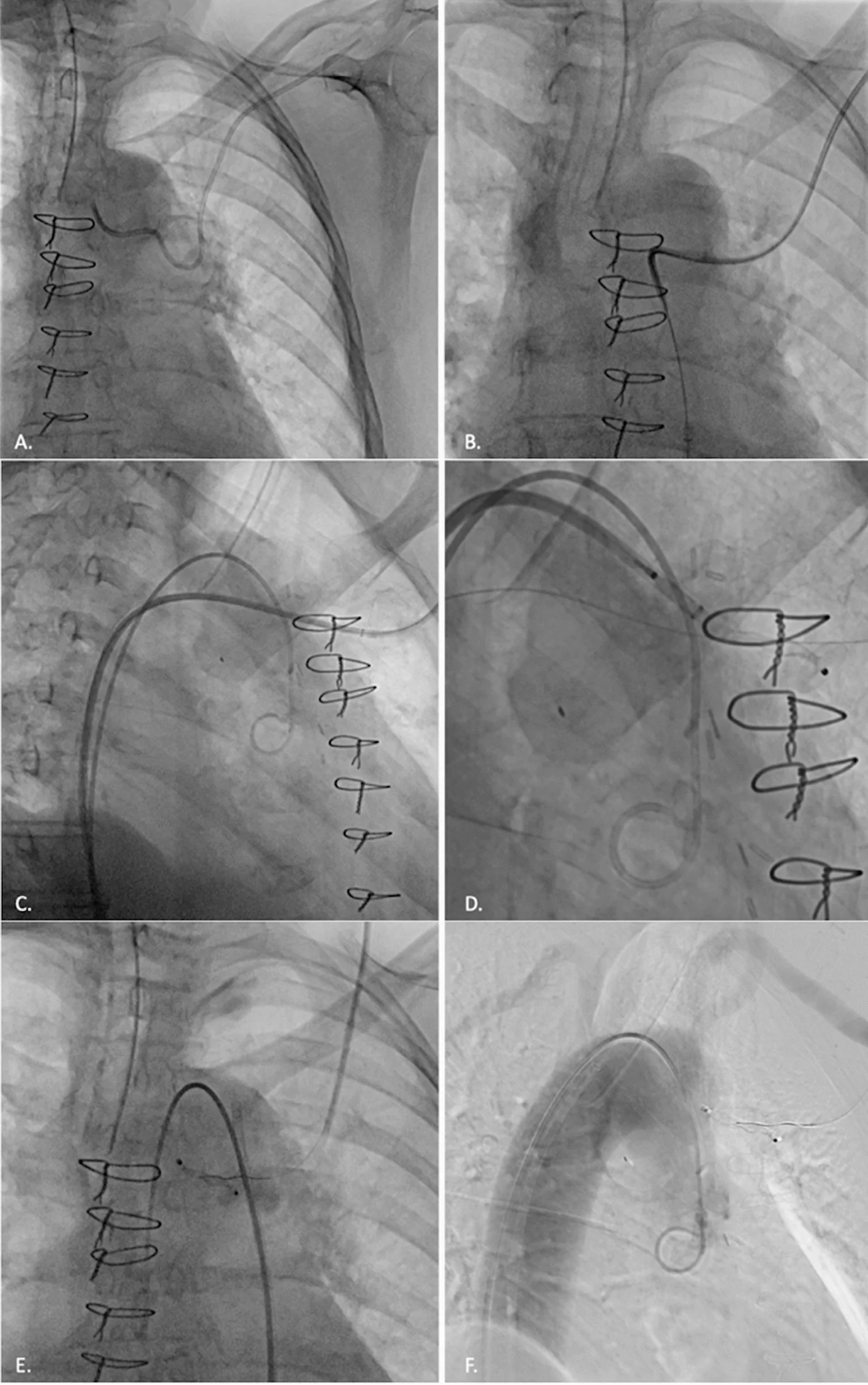
Endovascular retrieval of malpositioned catheter and tract embolization. **A** Fluoroscopic imaging showing a left-sided central venous catheter extending into the thoracic aorta **B** Establishment of a through-and-through wire access. **C** Advancement of a femoral sheath across the aortic arch perforation site. **D** Deployment of a 12-mm Amplatzer Vascular Plug II after sheath withdrawal to occlude the tract. **E** Retained 0.014’’ guidewire to facilitate potential additional embolization via microcatheter. **F** Final aortography demonstrating complete closure without contrast extravasation

After removal of the 0,035’’ guidewire, the vascular plug was advanced through the 7F sheath to its distal tip. Controlled sheath retraction enabled deployment across the entry site, with the distal portion positioned extraluminally and the proximal portion within the aortic lumen, effectively sealing the tract and anchoring the device across the aortic wall. The 0.014’’ guidewire was temporarily retained to allow additional embolization via microcatheter if needed. Final angiography confirmed absence of contrast extravasation, after which all wires were removed (Fig. 2). The right femoral access site was closed using a FemoSeal device, and the contralateral sheath was used for hemodynamic monitoring.

**Fig. 3:**
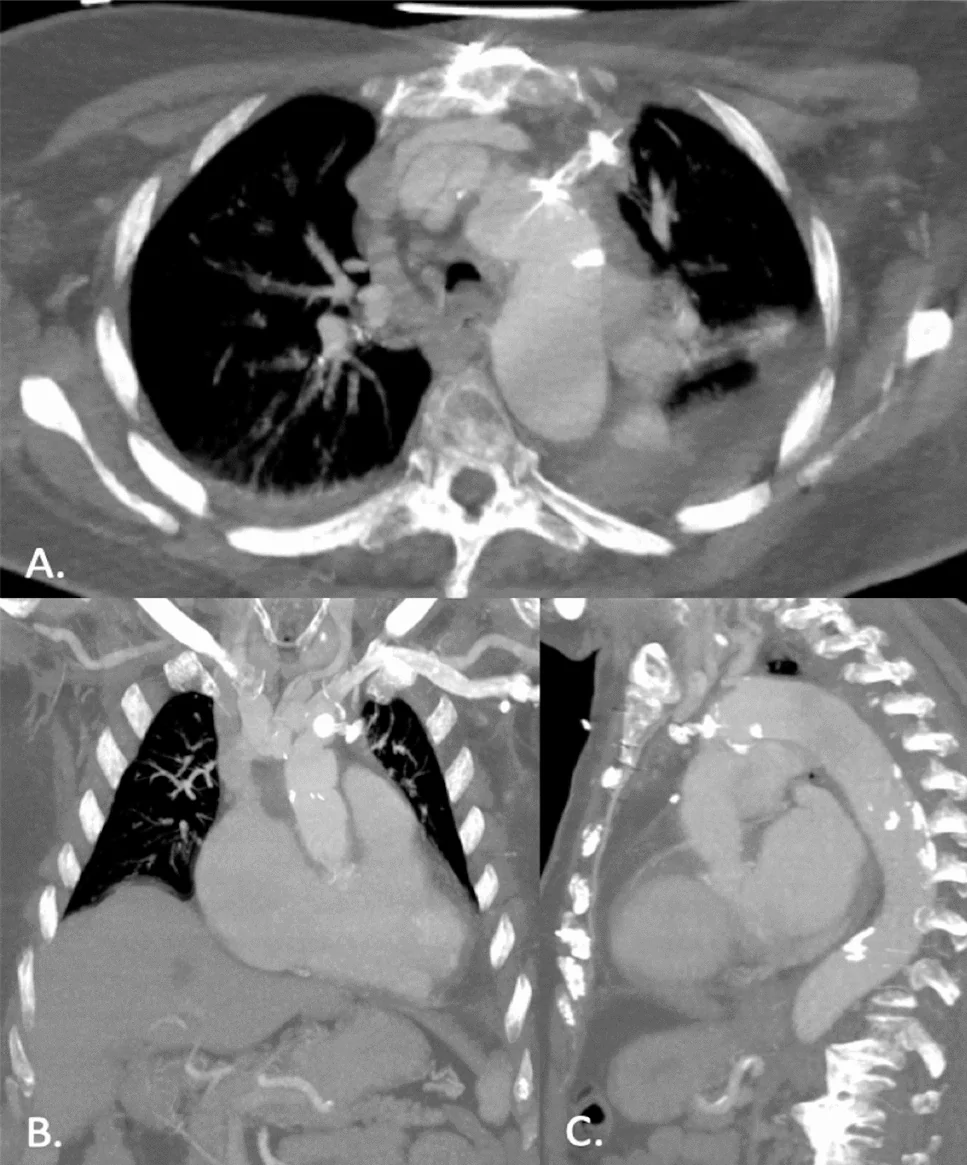
Post-interventional CT confirming successful catheter removal. Post-interventional contrast-enhanced CT in axial (**A**), coronal (**B**), and sagittal (**C**) planes with maximum intensity projection (MIP) reconstruction. The aortic arch tract is sealed following endovascular plug embolization, with no evidence of acute complications

Postoperatively, the patient was monitored in the ICU. CT imaging two days later confirmed complete catheter removal without hemorrhage or complications (Fig. 3). The patient was transferred to the general ward and discharged in stable condition shortly thereafter.

Central venous catheterization is associated with mechanical complications including arterial cannulation, which occurs in approximately 1.6% of cases [2]. Malposition into the aortic arch is extremely rare but associated with significant morbidity. Blind catheter removal is contraindicated due to high complication rates, including hemorrhage, pseudoaneurysm, stroke, and death [3]. Current management favors endovascular techniques over open surgery due to lower morbidity [4, 5]. However, the optimal approach depends on anatomical factors, catheter characteristics, and patient comorbidities, and requires careful procedural planning with appropriate imaging.

In the present case, conventional approaches were not feasible. External compression was impossible due to the intrathoracic location. Standard closure devices were unsuitable because of limited reach and inability to seal a high-flow aortic defect. Stent–graft placement was precluded by the absence of adequate landing zones near the supra-aortic vessel reimplantation sites. A septal occluder device was considered but rejected due to lack of suitable anchoring zones in this irregular, non-septal tract. In contrast, the Amplatzer Vascular Plug allowed better adaptation to the focal aortic defect and enabled controlled occlusion of the irregular pre-aortic/intrathoracic tract.

The vascular plug enabled controlled closure of the catheter tract with immediate hemostasis, avoiding complex reconstruction. Despite concomitant septicemia, the procedure was completed successfully without immediate or procedure-related complications, supporting its feasibility in carefully selected high-risk patients. Earlier contralateral access may have facilitated immediate angiographic confirmation and represents a potential procedural refinement.

Inadvertent aortic catheterization is a rare but life-threatening complication. When conventional surgical or stent-based approaches are unsuitable, endovascular plug embolization represents a safe and effective alternative, as demonstrated in this case.

## Abbreviations

CT: Computed Tomography
CVC: Central Venous Catheter
ICU: Intensive Care Unit
